# Nonlinear Hysteresis Parameter Identification of Piezoelectric Actuators Using an Improved Gray Wolf Optimizer with Logistic Chaos Initialization and a Levy Flight Variant

**DOI:** 10.3390/mi16050492

**Published:** 2025-04-23

**Authors:** Yonggang Yan, Kangqiao Duan, Jianjun Cui, Shiwei Guo, Can Cui, Yongsheng Zhou, Junjie Huang, Geng Wang, Dengpan Zhang, Fumin Zhang

**Affiliations:** 1 School of Mechanical and Power Engineering, Henan Polytechnic University, Jiaozuo 454003, China; 2 National Institute of Metrology, Beijing 100029, China; 3 Tianjin Jinhang Technical Physics Institute, Tianjin 300308, China; 4 Key Laboratory of Testing Technology for Manufacturing Process of Ministry of Education, Southwest University of Science and Technology, Mianyang 621010, China; 5 State Key Laboratory of Precision Measuring Technology and Instruments, Tianjin University, Tianjin 300072, China; zhangfumin@tju.edu.cn

**Keywords:** piezoelectric hysteresis, Bouc–Wen model, parameter identification, GWO

## Abstract

Piezoelectric tilt mirrors are crucial components of precision optical systems. However, the intrinsic hysteretic nonlinearity of the piezoelectric actuator severely restricts the control accuracy of these mirrors and the overall performance of the optical system. This paper proposes an improved Gray Wolf Optimization (GWO) algorithm for high-accuracy identification of hysteresis model parameters based on the Bouc–Wen (BW) differential equation. The proposed algorithm accurately describes the intrinsic hysteretic nonlinear behavior of piezoelectric tilt mirrors. A logistic chaotic mapping method is introduced for population initialization, while a nonlinear convergence factor and a Levy flight strategy are incorporated to enhance global search capabilities during the later stages of optimization. These modifications enable the algorithm to effectively identify BW model parameters for piezoelectric nonlinear systems. Compared to conventional Particle Swarm Optimization (PSO) and standard GWO, the improved algorithm demonstrates faster convergence, higher accuracy, and superior ergodicity, making it a promising tool for solving optimization problems, such as parameter identification in piezoelectric hysteresis systems. This work provides a robust approach for improving the precision and reliability of piezoelectric-driven optical systems.

## 1. Introduction

In recent years, the fast tilt mirror (FTM) has been an important and integrated opto-mechanical–electrical device for rapid and precise laser beam steering, and it is being employed more and more in the fields of optical signal transmission, adaptive optics, high-precision tracking control [1,2], and so on. Particularly, piezoelectric FTM (PFTM) systems leveraging piezoelectric actuators have gained prominence due to their exceptional advantages, such as their high displacement resolution (sub-nanometer scale), ultrafast response (microsecond-level), substantial driving force, high resonance frequency, and light weight. In principle, by using a piezoelectric actuator to generate a force to control one of its own flat mirrors, the laser beam is quickly and precisely adjusted by slight angles, enabling precise control and positioning of the incidence and exit light paths [3]. By employing piezoelectric elements to dynamically adjust mirror orientation, PFTMs are widely utilized in adaptive optics, target pointing, composite axis precision tracking, laser beam stabilization systems, astronomical telescopes, lidar, laser beam adjustment, and space optical communication [4,5,6,7,8,9,10].

However, the inherent hysteresis and creep phenomena of the piezoelectric material itself introduce significant nonlinearity between input voltage and output displacement. Also, the output of the PFTM presents a nonlinear mapping issue in practical applications, which has a significant impact on the system’s ability to regulate the PFTM’s speed and accuracy, and it further impacts the performance of the entire optical system. To gain linearization without taking into account the effect of hysteresis, while traditional closed-loop control strategies can mitigate these effects, they typically reduce the bandwidth of the tracking system, and they are only appropriate for applications with low dynamic performance requirements. This fundamental limitation underscores the critical need for advanced hysteresis modeling and compensation techniques to achieve both high precision and wide bandwidth in next-generation PFTM systems.

Current research on hysteresis modeling presents multiple approaches, each with distinct advantages and limitations. And, many scholars have conducted in-depth studies and developed various hysteresis models [11,12], including the Preisach model [13,14], the Krasnosel’skii–Pokrovskii (KP) model [15], the Prandtl–Ishlinskii (PI) model [16], the BW model [17,18,19], the Maxwell model [20], etc. The Preisach model, despite its comprehensive framework, requires identification of many parameters through extensive experimental data; the KP/PI models demonstrate limited adaptability to asymmetric hysteresis loops and dynamic frequency dependencies; and the Maxwell model, while effective for linear viscoelasticity, fails to capture the closed hysteresis loop behavior of nonlinear hysteresis, leading to inaccuracies in predicting cyclic loading responses. In contrast, the BW model offers a favorable balance with its few parameters, strong dynamic adaptability, and easy identification. This parametric efficiency makes BW modeling particularly suitable for real-time control applications, motivating its adoption in this study.

The parameter identification process for BW models presents its own challenges. Conventional approaches have been used for parameter identification of the BW model, such as the neural networks (NNs) method [21], genetic algorithms (GAs) [22], and PSO [23] algorithms. NNs require careful architecture design (typically, three to five hidden layers with 10–20 nodes) and extensive training data (>10^4^ samples), while GAs suffer from premature convergence (up to 40% probability in 30-dimensional spaces). PSO, though simpler to implement, exhibits sensitivity to initial conditions with solution variances exceeding 15% across runs. Consequently, it is difficult to achieve the characteristics of fast convergence, high accuracy, simple operation, and stability while using the above methods to identify model parameters. To date, there have been many studies on the application of the GWO [24] algorithm to parameter identification of piezoelectric hysteresis models [25] due to its simple structure, fast convergence, and high solution accuracy.

In this work, the improved GWO (IGWO) algorithm is applied to BW hysteresis model identification to explore its feasibility. Inspired by the Cuckoo Search (CS) algorithm [26], we attempted to extend the Levy flight variation of the CS updating mechanism into the GWO algorithm and found that it could significantly improve the optimization capability of the classical GWO algorithm. For the algorithm’s improvement, we verified the relevant initial parameter values through a series of vector experiments. Compared with PSO and GWO algorithms, the IGWO algorithm shows great enhancement in stability, accuracy, and ergodicity, and it can be applied to the identification of the BW model.

The rest of the paper is organized as follows. Section 2 presents the BW model used in this work, the classical GWO algorithm, and the details of the GWO algorithm’s improvements. Section 3 and Section 4 gives the results of the initial parameter setting and parameter identification and the performance comparison of the IGWO algorithm with the classical PSO and GWO. In Section 5, we offer conclusions for this work.

## 2. BW Model and GWO Algorithm Improvements

### 2.1. Description of the BW Model

The BW model can be used to describe the connection between the input voltage and the output angle in PFTM research. The model was proposed by Bouc [27] in 1967 and refined by Wen [28] in 1976 to demonstrate classical piezoelectric hysteresis properties [29]. With few parameters and a straightforward structure, the BW operator has been shown [30] to describe hysteresis nonlinear mapping phenomena that are symmetric in the beginning, which is widely used in a variety of systems for hysteresis modeling. In this work, a simplified BW model proposed by scholars for the piezoelectric hysteresis nonlinear mapping problem is chosen [31], and the mathematical model is expressed as follows.

(1) $$x(t)=g(t)+h(t)=K_{v}u(t)+h(t),$$

(2) $$\dot{h}(t)=\alpha \dot{u}(t)-\beta \left|\dot{u}(t)\right|{\left|h(t)\right|}^{n-1}h(t)-\gamma \dot{u}(t){\left|h(t)\right|}^{n}$$

where x(t) is the output angle of the PFTM; g(t) is the linear angle; h(t) is the lagging angle; $K_{v}$ is the ratio of the output angle of the PFTM to the input voltage; u(t) is the input volt; $K_{v}u(t)$ is the linear angle component; $\dot{h}(t)$ is the first order derivative of the hysteresis angle of the PFTM; α, β, and γ are three constants that regulate the shape of the hysteresis curve; $K_{v}$, α, β, and γ are unknown and need to be identified through an algorithm; and *n* is usually set as 1.

### 2.2. Description of Classical GWO Algorithm

The GWO algorithm, proposed by Mirjalili in 2014, is inspired by the leadership hierarchy and hunting behavior of gray wolves in nature. In the algorithm, a wolf population is classified into four groups, α, β, δ, and ω, based on a pyramidal structure from the highest to the lowest. The algorithm selects the top three optimal solutions as the α, β, and δ elite wolves and then drives the other ω wolves to continuously update their own positions according to the elite wolves’ positions, thus seeking the optimal solution. For the sake of presentation, we briefly describe the main steps.

#### 2.2.1. Initialization

The traditional GWO algorithm generates an $N_{p}$ *d*-dimensional target individual by initializing randomly, $P=\{X_{1,t},\;X_{2,t},\ldots \ldots ,X_{Np,T}\}.$ *N_p_* represents the size of the population, **P** stands for current position, *t* is the number of current iterations, and T is the maximum number of iterations, where $X_{i,T},=1,2,3,\cdots ,T$ represents the *i*-th individual in the search space $\left\{l_{b},u_{b}\right\}$, $l_{b}=\left\{\;{X^{1}}_{\min},{X^{2}}_{\min},\ldots ,{X^{d}}_{\min}\right\}$, and $u_{b}=\left\{\;{X^{1}}_{\max},{X^{2}}_{\max},\ldots ,{X^{d}}_{\max}\right\}$. The initialization is expressed as follows.

(3) $$P=rand(N_{p},d)\cdot (u_{b}-l_{b})+l_{b},$$

After initialization of the target individual, the next three steps are encirclement, hunting, and attacking. The location information is continuously updated until the optimal solution is finally found.

#### 2.2.2. Encirclement

In the course of encircling its prey, the gray wolf first gradually approaches its target and then encircles it, with the following mathematical model:

(4) $$\begin{array}{l} D=\left|C\cdot X_{p}(t)-X(t)\right|\\ X(t+1)=X_{p}(t)-A\cdot D \end{array},$$

where **A** and **C** are the coefficient vectors; **D** is the distance between the gray wolf and the prey; and *t* is the number of current iterations. **X***_p_* denotes the current position vector of the prey and **X** indicates the current position vector of a gray wolf.

The values of the vectors **A** and **C** can be calculated as the following two equations:

(5) $$\begin{array}{l} A=2a\cdot rand_{1}-a\\ C=2\cdot rand_{2} \end{array},$$

Here, **rand**_1_ and **rand**_2_ are one-dimensional random vectors in [0, 1], and components of **a** represent the convergence factors, expressed in Equation (6).

(6) a=2(1−t/T),

where *t* is the current iteration, *T* is the maximum number of iterations, and the convergence factor **a** decreases linearly from 2 to 0 over the number of iterations.

#### 2.2.3. Hunting

In this process, *α*, *β,* and *δ* elite wolves (solution vectors) with strong searching ability lead *ω* wolves to gradually approach the prey. *ω* wolves update their positions randomly under the guidance of the three elite wolves. Thus, the specific mathematical model is expressed as follows:

(7) $$\begin{array}{l} D_{\alpha }=\left|C_{1}\cdot X_{\alpha }-X\right|\\ D_{\beta }=\left|C_{2}\cdot X_{\beta }-X\right|\\ D_{\delta }=\left|C_{3}\cdot X_{\delta }-X\right| \end{array},$$

(8) $$\begin{array}{l} X_{1}=X_{\alpha }-A_{1}(D_{\alpha })\\ X_{2}=X_{\beta }-A_{2}(D_{\beta })\\ X_{3}=X_{\delta }-A_{3}(D_{\delta }) \end{array},$$

(9) $$X(t+1)=\frac{X_{1}+X_{2}+X_{3}}{3},$$

**D**_α_, **D**_β_, and **D**_δ_ in Equation (7) denote the magnitude of the distances from *α*, *β*, and *δ* to the *ω* wolf, respectively; **X**_1_, **X**_2_, and **X**_3_ indicate the position vectors of the *α*, *β,* and *δ* wolves at the current number of iterations, respectively.

#### 2.2.4. Attacking

During the course of attacking the prey, the gray wolf adjusts the distance between the prey and the gray wolf mainly through fluctuation of the range of **A**. When random values of **A** are in [−1, 1], the gray wolf continues the search operation, and it attacks the prey to complete the hunt outside of the enclosure.

### 2.3. GWO Algorithm Improvement

The dynamic input–output characteristics of PFTM are significantly influenced by the hysteresis effect inherent in the piezoelectric actuator. The BW model can describe this hysteretic nonlinear behavior well. However, the accuracy of the model and its correspondence with the actual hysteresis curve are highly dependent on the values of the unknown parameters within the model. The traditional GWO algorithm, while effective in many optimization tasks, often struggles with local optima and lacks the ability to converge quickly and stably when dealing with nonlinear, unknown parameters. The following improvements have been made to resolve these issues, and they are applied to piezoelectric hysteresis parameter identification.

#### 2.3.1. Logistic Chaos Initialization

Traditional meta heuristic algorithms perform population initialization by randomly generating populations of individuals, which makes it difficult to make the population more diverse. And, the non-diverse characteristic easily leads to unstable wolf species, which directly influences the solution efficiency and accuracy of the algorithm. Considering the randomness, diversity, and regularity of logistic chaotic mapping initialization, the chaos is generated, and it is also one of the unique dynamical sources of nonlinear systems. It is a special substance between specificity and randomness. We employ it in the GWO algorithm to replace the random initialization. It can not only enrich the wolfpack’s diversity but also lead the algorithm to jump out of the local apices well so as to avoid falling into prematureness. It will improve the solution speed and thus obtain a global optimal solution. The chaos initialization equation is expressed as follows.

(10) $$P=F\cdot rand(N_{P},d)\cdot (1-rand(N_{P},d)),$$

where the adjustment factor **F** in chaos initialization typically ranges from 2 to 4.

#### 2.3.2. Convergence Factor Improvement

In the GWO algorithm, the convergence factor **a** is the one that decreases linearly as the algorithm converges. This convergence does not deal well with nonlinear problems and balances the local and global search capabilities of the algorithm, which affects the efficiency of the algorithm’s solution and convergence accuracy. Mitta N et al. [32] proposed the use of an exponential approach to adjust the previous linear function, which could be a good solution to the engineering problem. According to the problem that the GWO algorithm is prone to fall into local optimum, the convergence factor *a* is adjusted to be a nonlinear convergence factor to fine-tune the convergence speed of the algorithm in the early stage to strengthen the local search ability of the algorithm, and then the convergence speed is accelerated in the later stage to balance the global search ability of the GWO algorithm and improve the convergence of the algorithm. The specific mathematical form is expressed as follows:

(11) a=2(1-exp(t/T)),

#### 2.3.3. Levy Flight Variant

The search accuracy of the GWO algorithm is lower, and its stability is weaker. To address these limitations and enhance the algorithm’s performance, inspired by the Cuckoo Search algorithm, we incorporate Levy flight variation [33] into the GWO algorithm on the basis of the original search method. The Levy flight variant is of good effect in complementing the local search capability, the search accuracy, and the stability of the algorithm, and it improves the performance of the presented algorithm. The mathematical formation of the Levy flight variant is expressed in Equation (12).

(12) $$\begin{array}{l} X_{1}=X_{1}+\Gamma Levy(s)\\ X_{2}=X_{2}+\Gamma Levy(s)\\ X_{3}=X_{3}+\Gamma Levy(s) \end{array},$$

(13) $$s=u/{\left|v\right|}^{1/\beta },$$

(14) $$X=\frac{X_{1}+X_{2}+X_{3}}{3},$$

In recent years, many researchers have conducted in-depth studies on Levy(s), where Γ is the step size coefficient, Levy(s) is the random path strategy, and *s* is a good way to model Levy flight [34] random steps proposed by Mantegna in 1994. Where

(15) $$u\sim N(0,\sigma^{2}),v\sim N(0,1),\sigma ={\left\{\left.\frac{\Gamma (1+\beta )\sin(\frac{\pi \beta }{2})}{\beta \Gamma (\frac{1+\beta }{2})2^{\frac{\beta -1}{2}}}\right)\right.}^{\frac{1}{\beta }},$$

#### 2.3.4. Algorithm Workflow

The algorithm iteratively updates its parameters from initialization until it converges to the optimal fitness function. As shown in Algorithm 1, the pseudo-codes for the improved GWO process are outlined as follows.**Algorithm 1.** The improved GWO.1:BEGIN2:Initialization of gray wolf population with Equation (3)3:Initialization of **a**, **A,** and **C**4:Calculate the fitness function for each search agent5:Assume *X*_α_ for the best search agent, *X*_β_ for the second search agent, and *X*_δ_ for the third search agent6:While *t* < = T do7:For each search agent8:Update the **a**, **A,** and **C**9:$\mathrm{If}\;\left|A\right|<1$10:Update the position of the current search agent using Equations (10), (12) and (14)11:$\mathrm{If}\;\left|A\right|>1$12:Update X if there is a better solution13:End for14:Check if any search agent goes beyond the search space and amend it15:Calculate the fitness of all search agents16:*t* = *t* + 117:End while18:BEGIN19:END

### 2.4. Principles of Parameter Identification

**Figure 1:**
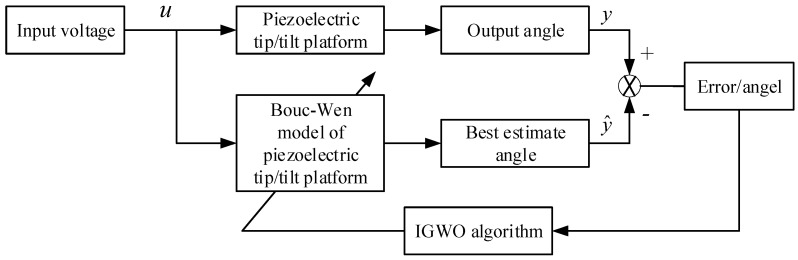
Principle of parameter identification.

Figure 1 shows the principle of parameter identification. The input voltage u is separately applied to the piezoelectric tip/tilt platform and its BW model. The output angle y of the tip/tilt platform is collected continuously, and the best estimate angle $\hat{y}$ of the model is simultaneously calculated. Thus, there exist the errors between them. The IGWO algorithm proposed in the paper is employed to identify the parameters of the BW hysteresis nonlinear model of the piezoelectric tilting mirror, and the estimate angle $\hat{y}$ can be updated in real time. By comparing it with the output angle of the experimental platform, the accuracy of the model will be improved constantly. Here, we take Relative Mean Squared Error (RMSE) as a benchmark for the fitness function *J,* as presented in the following mathematical Equation (16).

(16) $$J(k_{v},\alpha ,\beta ,\gamma )=\sqrt{{\sum_{i=1}^{n}\left(Y_{e}^{i}-Y_{m}^{i}\right)}^{2}/\sum_{i=1}^{n}(Y_{m}^{i}{)}^{2}},$$

where $Y_{e}^{i}$ is the actual PFTM deflection angle at each moment; $Y_{m}^{i}$ is the output angle of the model at each moment; *n* is the size of sampled data; and *J* is an adaptation function containing four unknown parameters and its value determines the model’s accuracy.

## 3. Experimental Verification

### 3.1. Experimental Platform Construction

**Figure 2:**
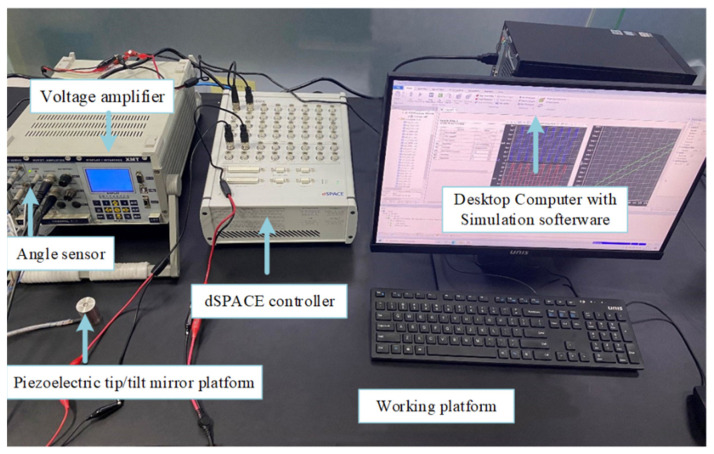
Experimental platform.

Figure 2 shows the experimental platform layout of this study. The experiment setup mainly includes a working platform, a desktop computer, a dSPACE controller with A/D and D/A interface, a piezoelectric tip/tilt mirror platform, a set of voltage amplifiers, and an angle sensor (controller model: XE501-D; piezoelectric ceramic driver model: XS330.2SL, made from Harbin Core Tomorrow Science & Technology Co., Ltd., Harbin, China).

**Figure 3:**
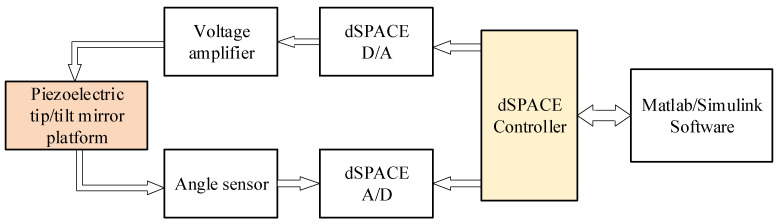
Experimental process structure.

Of them, the computer is used to build the output excitation source in the MATLAB/Simulink R2022b environment using a Windows system. The dSPACE controller can play an important role in adjusting the parameters online and in real time, and it sends a voltage signal through the D/A interface. The output angle data of the PFTM, collected by the angle sensor, are transmitted back through the A/D interface. The voltage amplifier amplifies the voltage signal from the dSPACE by a magnification of 12 times. The experimental process is shown in Figure 3. 

### 3.2. Selection of Algorithm Parameters

At the beginning phase of the algorithm, we set the upper and lower boundaries uniformly, as follows: $l_{b}=[0.1,0.1,0.1,0.1],u_{b}=[10,10,10,10]$. Other parameters, such as the iteration number T and the population size Np, need to be verified through a series of experiments.

**Figure 4:**
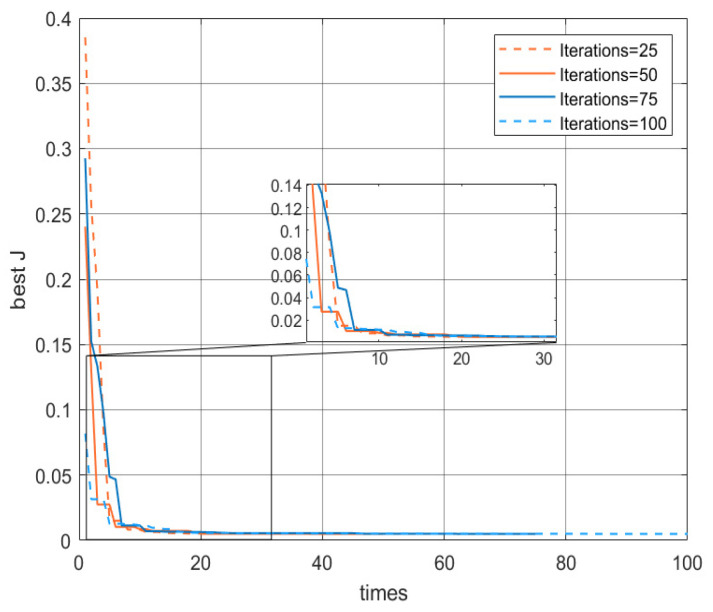
Comparison of the number of iterations.

To determine the maximum iteration number, we set 25, 50, 75, and 100 as the maximum iteration number in each of four conditions. The population size Np is set to 40, which is 10 times the 4 unknown parameters, and the chaos factor F is taken as an initial value of 2. As shown in Figure 4, the IGWO algorithm can reach convergence quickly, and the convergence results are relatively stable. Their optimal fitness values are 0.00506, 0.00491, 0.00491, and 0.00493. Taking into account the time factor and the number of iterations, we determine the iteration number T = 50.

**Figure 5:**
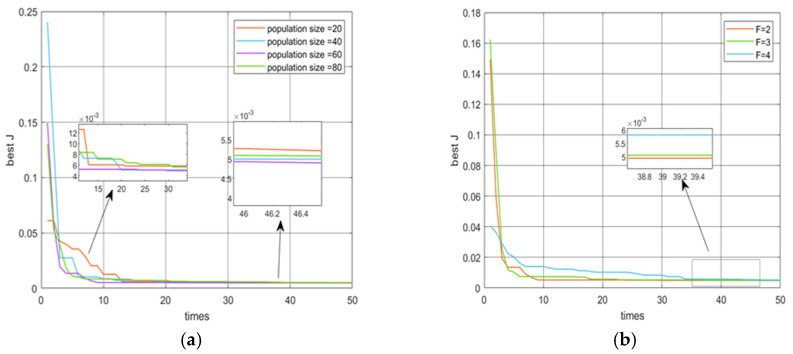
The curves of fitness function *J* with different parameters. (**a**) Comparison of best J with population size; (**b**) iterative comparison of best J with the values of the chaos factor F.

After the iteration number T is determined to be 50, we discuss the effect of the population size on the fitness function by setting the population size Np to be 20, 40, 60, and 80, with the constant chaos factor F = 2. As shown in Figure 5a, the corresponding fitness functions were 0.00514, 0.00491, 0.00486, and 0.00504. Therefore, the population size N_p_ is 60. In a word, T for 50 and N_p_ for 60 are determined correctly. Then, we discuss whether the size of the chaos factor F for chaos initialization affects the size of the fitness value. As can be seen from Figure 5b, the effect of F is still significant, with the values of 2, 3, and 4 of the chaos factor F on the fitness functions used being 0.00486, 0.00493, and 0.00519, respectively. Finally, we choose the chaos factor F to be 2 as the chaos initialization value.

### 3.3. Parameter Identification Results

**Figure 6:**
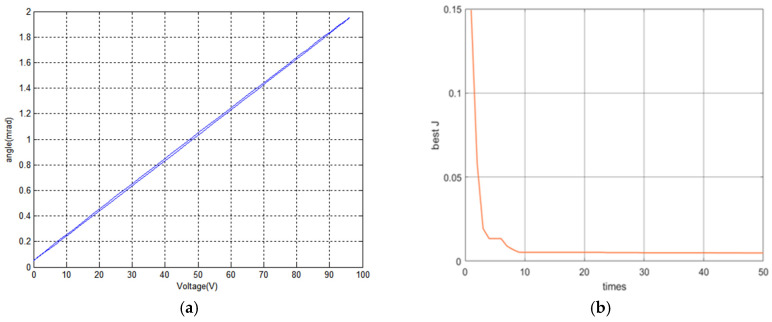

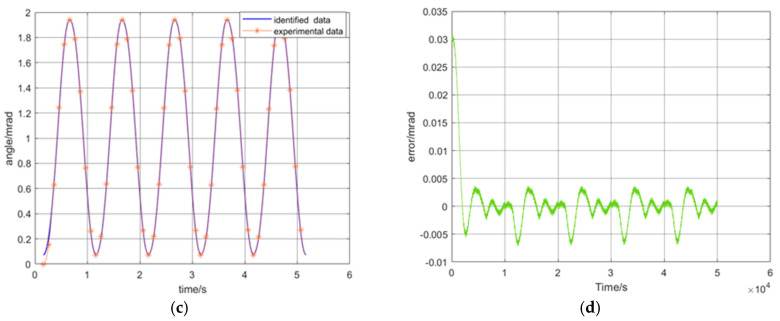
IGWO identification results. (**a**) input voltage and out angle; (**b**) IGWO iteration curve; (**c**) experimental output angles and identification output data; (**d**) error curve.

After all of the parameters have been determined successfully, the input voltage is set for U=48sin(2πft−2.5)+48, here *f* = 1. Thus, we can obtain the output data of the PFTM and the identification results of the BW model using the IGWO algorithm. Figure 6a shows the relationship between input voltage and out angle; Figure 6b shows the iterative curve of the fitness function; Figure 6c shows the angle tracking curve; and Figure 6d shows the output error curve of the piezoelectric tilt mirror compared with the BW model.

**Figure 7:**
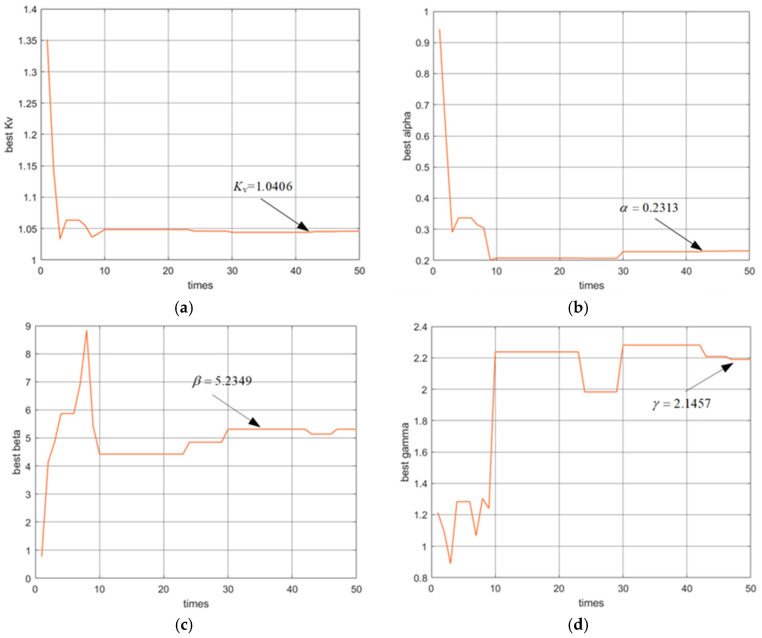
Convergence history of unknown parameters. (**a**) Convergence history of $K_{v}$; (**b**) convergence history of α; (**c**) convergence history of β; (**d**) convergence history of γ.

The four parameters are obtained successfully while the values trend toward stability. The convergence curve history of the four unknown parameters, $K_{v},\alpha ,\beta ,\gamma$, is approximately 1.0406, 0.2313, 5.2349, and 2.1457, as shown in Figure 7.

The values of the unknown parameters with the BW model for the PFTM are listed in row 2 of Table 1. The results indicate that the IGWO algorithm is better employed in the parameter identification of the hysteresis model of PFTM.

### 3.4. Comparative Analysis of Three Algorithms

**Figure 8:**
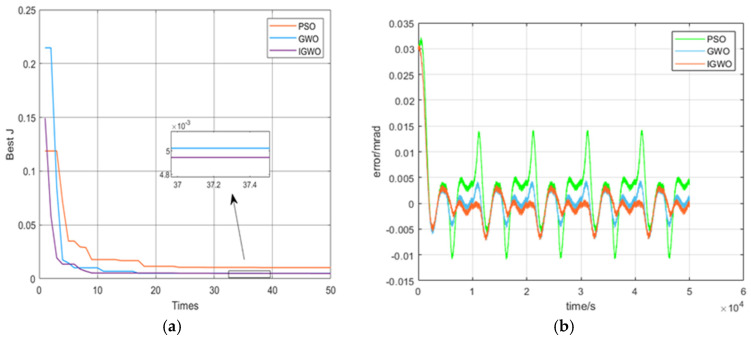
Performance comparison for PSO, GWO, and the proposed IGWO in this paper. (**a**) Algorithm iteration comparison; (**b**) error comparison of different algorithms.

To prove the IGWO’s performance, the classical GWO algorithm and the PSO algorithm are also used in parameter identification to describe its good performance in this study. In the experiments, the same initialization parameters, such as upper and lower bounds and fitness functions, are guaranteed to be set consistently. We can see that the IGWO has good performance advantages in terms of iteration speed, convergence accuracy, and error profile, as shown in Figure 8.

As seen from Figure 8a, the IGWO algorithm has a significant accuracy difference compared to the classical PSO algorithm in terms of the output angle fitted to the model. The value of the fitness function J is approximately $4.8\times 10^{-3}$ for IGWO, while the value is approximately $7.1\times 10^{-3}$ for PSO. As seen from Figure 8b, the error for IGWO is lower than that of GWO and PSO. The results show that the accuracy of the IGWO presented in this study is better than that of GWO and PSO. The specific fitness function values and the values of the unknown parameters are shown in Table 1.

**Figure 9:**
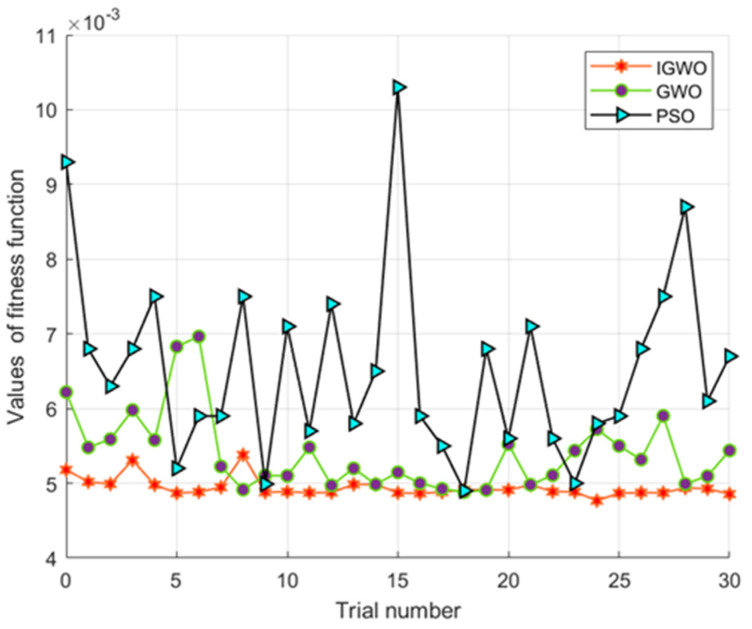
Comparison of different algorithms for 30 experiments.

Although the comparison cannot fully reflect the excellent performance of the IGWO, we subjected the three algorithms to initialization experiments with different population sizes. Thirty groups of experiments were conducted for each algorithm, and Figure 9 shows a comparison of the fitness function values using IGWO, GWO, and PSO. Also, the specific minimum, maximum, and average fitness function values are shown in Table 2.

## 4. Discussion

The IGWO, enhanced by Levy flight and logistic chaos initialization, demonstrates significant performance improvements in the parameter identification of hysteresis nonlinearity in piezoelectric actuators. As seen in Table 2, the specific minimum, maximum, and average fitness function values of the IGWO are lower than those of the other two. Certainly, as seen from Figure 9, the fitness degree of the GWO is developed greatly, while the traditional PSO and GWO algorithms tend to fall into local minima, which occurred several times during the course of conducting the experiments. We can also see that the improved GWO algorithm has very good accuracy and stability, and it competes well with the other two algorithms.

Despite the performance improvements, the proposed algorithm still has some limitations, like computational complexity, parameter sensitivity, etc. To further enhance the performance and applicability of the parameter identification algorithm, future research can focus on the following aspects: (1) explore more efficient chaos mapping methods or hybrid optimization strategies to further enhance the accuracy of parameter identification; (2) reduce computational complexity through parallel computing or adaptive parameter adjustment mechanisms to improve runtime efficiency; and (3) conduct in-depth analysis of the mechanisms through which Levy flight and chaos initialization affect algorithm performance, providing theoretical guidance for parameter settings.

## 5. Conclusions

In this study, the BW model and the hysteretic nonlinear behavior of the PFTM were thoroughly investigated. An IGWO algorithm was proposed to accurately identify the four unknown parameters of the BW model, which characterizes the hysteretic nonlinearity of the PFTM. The IGWO algorithm incorporates logistic chaotic population initialization, a nonlinear convergence factor, and a Levy flight variant, significantly enhancing its convergence speed, search accuracy, stability, and traversal capabilities. Comparative experiments with the traditional PSO algorithm and the classical GWO algorithm demonstrate the superior performance of the proposed IGWO.

This work provides a robust and efficient intelligent optimization approach for identifying hysteretic model parameters in piezoelectric nonlinear systems, offering valuable insights for precision control applications.

## Figures and Tables

**Table 1:** Parameter identification results and fitness function value.

Algorithm Type	*J*	$K_{v}$	α	β	γ
IGWO	$4.8\times 10^{-3}$	1.0406	0.2313	5.2349	2.1457
GWO	$5.2\times 10^{-3}$	1.0457	0.2249	6.8862	0.5358
PSO	$7.1\times 10^{-3}$	1.0402	0.4070	7.2397	7.4774

**Table 2:** Performance comparison of the different algorithms.

Algorithm Type	IGWO	GWO	PSO
Min *J*	$4.76\times 10^{-3}$	$4.88\times 10^{-3}$	$4.90\times 10^{-3}$
Max *J*	$5.38\times 10^{-3}$	$6.96\times 10^{-3}$	$1.03\times 10^{-2}$
Average value *J*	$4.96\times 10^{-3}$	$5.63\times 10^{-3}$	$6.52\times 10^{-3}$

## Data Availability

The original contributions presented in this study are included in the article. Further inquiries can be directed to the corresponding author.

## Abbreviations

The following abbreviations are used in this manuscript:

GWO	Gray Wolf Optimization
IGWO	Improved Gray Wolf Optimization
BW	Bouc–Wen
PSO	Particle Swarm Optimization
FTM	Fast tilt mirror
PFTM	Piezoelectric fast tilt mirror
KP	Krasnosel’skii–Pokrovskii
PI	Prandtl–Ishlinskii
NN	Neural network
GA	Genetic algorithm
